# Evaluation of the Psychometric Properties of the Chinese Internet Gaming Disorder Checklist (C-IGDC) Among Chinese Adolescents

**DOI:** 10.3389/fpsyt.2021.721397

**Published:** 2021-09-13

**Authors:** Juliet Honglei Chen, Xiaoyu Su, Le Dang, Anise M. S. Wu

**Affiliations:** ^1^Centre for Cognitive and Brain Sciences, Institute of Collaborative Innovation, University of Macau, Macao SAR, China; ^2^Department of Psychology, Faculty of Social Sciences, University of Macau, Macao SAR, China; ^3^Faculty of Teacher Education, Pingdingshan University, Pingdingshan, China

**Keywords:** gaming disorder, adolescent, assessment, cutoff point, *DSM-5*, prevalence, Chinese, IGD

## Abstract

As the first *DSM-5* based, multidimensional screening tool of Internet gaming disorder (IGD) designed for Chinese gamers, the Chinese Internet Gaming Disorder Checklist (C-IGDC) has shown satisfactory psychometric properties among Chinese young adults. Given the high vulnerability to IGD among adolescents, the present study aimed to examine the applicability of C-IGDC to Chinese adolescents to address shortfalls in the existing literature regarding the assessment of adolescent IGD screening. Through a two-stage sampling method in a cross-sectional survey, we obtained a sample of 1,253 Chinese past-year adolescent gamers (43.8% female; *M*_*age*_ = 14.49 years, *SD*_*age*_ = 1.60 years) from local junior or senior high schools in Macao, China for data analysis. Our results confirmed a good model fit of the original two-level, nine-subfactor structure of the 27-item C-IGDC, and indicated adequate internal consistency and test–retest reliability, as well as good concurrent validity as evidenced by expected associations with IGD functioning impairments, gameplay characteristics, and depressive symptoms. An optimal screening cutoff score (≥20) was proposed to identify probable disordered gamers among Chinese adolescents with past-year gaming experience. The findings support the extended use of C-IGDC to Chinese adolescents as a reliable and valid assessment tool for evaluating IGD severity levels and screening for probable IGD cases. Its use can facilitate a proper screening procedure for probable IGD cases in both school and clinical settings.

## Introduction

Disordered Internet gaming is a widespread public health condition with grave danger to adolescents' wellbeing and development [for a review, see (1)]. A historically high prevalence of disordered gamers in Asian adolescents with the age of 12–20 years laid the foundation of the recognition of Internet Gaming Disorder (IGD) or Gaming Disorder (GD) as a mental/behavioral disorder in the fifth edition of the Diagnostic and Statistical Manual of Mental Disorders (*DSM-5*) (2) and the International Classification of Diseases 11th Revision (*ICD-11*) (3). In recent years, a significantly higher IGD prevalence still prevails regionally in Asia [e.g., a pooled 10.1%^1^ in southeast Asian adolescents and young adults (4)] compared to that observed on a global scope [e.g., a pooled 4.7%^2^ in the general population (5); a pooled 4.6%^3^ in adolescents (6)]. Taking a closer look into these pooled statistics, one cannot overlook the wide range of IGD prevalence estimates across various studies, such as 5.4–17.7% in Chia et al. (4), 0.7–15.6% in Feng et al. (5), and 0.6–19.9% in Fam et al. (6). Similarly, Chinese studies also reported varying adolescent IGD prevalence, ranging from 3.2–3.7% among secondary school students in a large-scale survey in the Chinese mainland (7) to 13.0–15.6% among secondary school students in Hong Kong (8, 9). Aside from sampling issues (e.g., convenient sampling vs. random sampling), another primary source attributed to such significant discrepancies in IGD prevalence is measurement issues, which include but are not limited to conceptual inconsistencies in diagnostic criteria of IGD (e.g., adapted from other addictive behaviors like Internet addiction and problematic gambling) and a lack of or insufficient psychometric evaluation of the IGD assessment tool in the corresponding population [for reviews, see (10, 11)].

These measurement issues are also commonly observed in adolescent IGD measures used in Chinese studies. Early indigenous assessment tools [e.g., (12–14)], constructed before the release of *DSM-5* and without a consensus on the IGD diagnostic criteria, were still frequently adopted in recent Chinese adolescent studies (15). The use of non-indigenously developed IGD scales in Chinese communities is also not uncommon in the current decade. However, some of those scales, such as Yu et al.'s (16) adaptation based on Gentile et al.'s (17) pathological video-game use scale, not only shared the same conceptualization problems of using non-IGD specific diagnostic criteria as the early Chinese IGD scales did, but also had some of the missing links in the psychometric evaluations (e.g., neglecting the assessment on the scale's dimensionality and latent factor structure). More recently developed *DSM-5* based IGD screening tools, like the ten-item Internet Gaming Disorder Test (18) and the Internet Gaming Disorder-20 Test (19), have solved the conceptualization issue by referring to the same set of IGD-specific diagnostic criteria. Although validated Chinese versions of these tests are available [e.g., (20, 21)], some nuanced indigenousness of Chinese gamers might be lost in the validation process because those items were not originally derived from the Chinese population. The abovementioned measurement issues may hamper accurate identifications of probable IGD cases, confound empirical investigations on IGD etiology and prognosis, and hinder timely preventions and interventions for high-risk adolescent gamers.

To address the inadequacy of Chinese adolescent IGD screening, the present study aimed to evaluate the psychometric properties of the indigenously-developed, *DSM-5* based Chinese Internet Gaming Disorder Checklist (C-IGDC) (22) among Chinese adolescents to provide a reliable and valid screening tool for probable adolescent IGD cases. The 27-item C-IGDC is the first *DSM-5* based, multidimensional IGD screening tool designed for Chinese gamers and has exhibited satisfactory reliability, validity, and screening efficacy for Chinese young adults (22). It adopts a two-level structure: a parent second-order C-IGDC construct represents the overall IGD severity, whereas the nine subordinating first-order subfactors entail the nine *DSM-5* diagnostic criteria (e.g., preoccupation and withdrawal). The optimal screening cutoff score for detecting probable IGD cases was set as ≥20, which corresponds to 81.7% sensitivity and 85.4% specificity in Chinese adults (22). In the present study, we attempted to assess the applicability of C-IGDC for Chinese adolescents by assessing its model structure, reliability, and validity, as well as proposing an optimal screening cutoff point for the Chinese adolescent population to facilitate an efficient mass screening for those who might need an additional diagnosis and support in clinical settings. According to mainstream findings on IGD epidemiology, disordered gamers would generally display multiple functioning impairments that jeopardize their physical and psychological wellbeings, interfere with schoolwork and relationships with family and friends, and cause subjective distress (23–26). These patterns were captured with three functioning impairment indicators in the current study as a part of the concurrent validity test for the adolescent C-IGDC; these indicators were also in accordance with the clinically significant impairment and/or distress caused by gameplay as proposed by the *DSM-5*'s (2) IGD diagnostic criteria. Other factors included for the concurrent validity testing are typical gameplay characteristics of disordered gamers, such as high investment in games in terms of time amount (1, 27, 28), gameplay frequency (22, 26), and monetary expense (9, 29, 30), as well as depressive symptoms, which were commonly observed among IGD gamers (26, 31, 32).

## Methods

### Participants and Procedures

The present study set the inclusive criteria as both sexes of Chinese adolescents who were receiving secondary schooling upon data collection and were able to read Chinese. A recommended minimum sample size of 200 was calculated with Soper's (33) a-priori sample size calculator for structural equation models with an anticipated medium effect size of 0.30 and a desired statistical power level of 0.80 according to Cohen's (34) guidelines. We started by inviting a random sample of local private and public secondary schools in Macao, China, to participate in the study at the first stage. For the second stage, we treated each class as a cluster within each school and asked the participating schools to randomly select a desirable number of clusters from different study grades according to the school size. In those chosen classes, trained research assistants briefed students about the study's details and its anonymous and voluntary nature and distributed copies of informed consent to students interested in our study beforehand. Upon formal data collection, we only collected data from students who met the criteria of (a) giving a written participation consent of their own and (b) not receiving an opt-out notice from their legal guardians. A similar opt-out design was adopted by a former large-scale longitudinal study among Chinese secondary students (35). The present study has obtained ethics approval from the affiliated institute of the corresponding author and conducted all the procedures following the latest version of the Declaration of Helsinki.

In the end, 12 schools and 86 classes took part in our study from late October to early December 2020. Among 1,476 students who completed the survey, 1,407 students' responses passed our validity check and hence were considered as valid cases (46.9% female, 95% CI [44.3%, 49.5%]; age: range = 11–18 years, *M* = 14.48, and *SD* = 1.60). To assess the test–retest reliability of C-IGDC, we also collected 246 matched cases (43.5% female, 95% CI [37.3%, 49.7%]; age: range = 11–18 years, *M* = 14.31, and *SD* = 1.72) from three schools 2 months later following the first data collection date. Because having gaming engagement within the past 12 months is a prerequisite for an IGD diagnosis (2), we applied an exclusion criterion of no gaming experience in the past 12 months, with a pre-screening question in this regard and hence ruled out 90 non-past-year gamers (6.4%). Furthermore, we excluded 64 cases (4.5%) with missing values due to the requirement of no missing data on any of the C-IGDC items for the psychometric properties analyses on C-IGDC. As a result, a final sample of 1,253 past-year gamers (43.8% female, 95% CI [41.1%, 46.5%]; age: range = 11–18 years, *M* = 14.49, and *SD* = 1.60) was generated for data analysis. In this past-year gamer sample, according to their self-report rating of socioeconomic class, most of them were from a middle-class family (63.0%), followed by 20.0% lower-middle, 10.1% upper-middle, 1.9% lower, 1.2% upper, and 3.8% missing.

### Measures

We requested participants to provide basic information of sex (1 = *male*, 2 = *female)*, age, grade level (from 1 to 6, corresponding to six grades of the secondary school), and socioeconomic status (from 1 = *lower* to 5 = *upper*). In addition to demographics, we also surveyed participants' past-year gameplay characteristics (including both online and offline gaming) and induced functioning impairments, as well as past-week depressive symptoms.

#### C-IGDC

The 27-item, 9-subfactor C-IGDC assessed how frequently the participants experienced each of the featured problematic gaming conditions in the past year on a 3-point scale, in which 0 = *never*, 1 = *sometimes*, and 2 = *often* (see Supplementary Table S1 for the C-IGDC in Chinese). A sample item is, “How often did you feel like you cannot stop Internet gaming (in the past 12 months)?” The summative item scores of a composite C-IGDC factor (i.e., total score) and its nine subfactors (i.e., mean score) were computed to indicate the extent of one's problematic gaming, with higher scores corresponding to greater severity. C-IGDC exhibited an acceptable range of reliability for the one composite factor (α = 0.92) and all the nine subfactors (α*s* = 0.65–0.79) when being tested in a Chinese young adult sample (22).

#### Diagnostic Criteria of IGD in the *DSM-5*

Participants endorsed whether or not they have undergone any of the nine *DSM-5* IGD diagnostic symptoms over the past 12 months (1 = *yes*, 0 = *no*), on items such as “Have you made unsuccessful attempts to control the participation in Internet games (in the past 12 months)?” The scale presented satisfactory reliability in this study (KR-20 = 0.67). Following the guidance of the *DSM-5*, we adopted a cutoff of *DSM-5* IGD total score ≥ 5 to classify probable IGD cases as a reference to determine an optimal screening cutoff point of C-IGDC for detecting probable IGD cases among Chinese adolescents that may need a further clinical diagnosis.

#### Gameplay-Induced Functioning Impairments

Three gameplay-induced functioning impairments, adapted from Lin et al. (36) proposed functioning impairment indicators for problematic smartphone use, were utilized to evaluate the existence of (a) physical or mental problems, (b) relations, study, and/or work impairments, and (c) mental distress that were shown due to one's gameplay. Participants responded to each single-item impairment indicator on a dichotomous response scale (1 = *yes*, 0 = *no*), with a sample item being, “Has your gameplay resulted in persistent or recurrent physical or psychological problems (in the past 12 months)?”

#### Gameplay Characteristics

Three additional items related to participants' gaming pattern in the past year were used, including (a) weekly gameplay amount estimation in hours, (b) weekly gameplay frequency (from 1 = *fewer than 1 day on average* to 5 = *almost every day*), and (c) monthly gameplay expense (11 points from 0 = *0 Patacas [0 USD]* to 11 = *10,000 Patacas [1,256 USD] or above*).

#### Depressive Symptoms

Depression symptoms were assessed by the 7-item depression subscale from the Chinese version of the 21-item Depression Anxiety Stress Scale (DASS-21) (37). Participants reported the frequency of their depressive symptoms, if any, experienced over the past week, on a 4-point Likert scale (0 = *did not apply to me at all* to 3 = *applied to me very much or most of the time*). A sample item is “I felt that I had nothing to look forward to.” A converted index score of depressive symptoms, ranging from 0 to 42, was computed using the total score of the seven items and then multiplied by two. A higher index score indicates more depressive symptoms. This subscale showed satisfactory reliability in the current study, with Cronbach's α = 0.88.

### Statistical Analyses

We utilized confirmatory factor analysis (CFA) to evaluate (a) whether the present adolescent data supported the original two-level, nine-subfactor model structure previously found in the Chinese adult sample [i.e., one second-order latent construct and nine first-order latent subfactors; for more details, see (22)], and (b) whether this hypothesized two-level model is superior than its single-factor alternative, more parsimonious model. As designed, we treated C-IGDC items as ordinal variables and specified a two-level CFA in Mplus 7.4 (38). The robust weighted least squares estimation mean and variance adjusted (WLSMV) method was chosen because it is a preferred estimation method for ordinal variables (39). We considered the goodness of model fit as satisfactory if it met the following criteria: CFI ≥ 0.95, TLI ≥ 0.95, RMSEA ≤ 0.08, and SRMR ≤ 0.08 (40, 41). The internal consistency and 2-month test–retest reliability were estimated by Cronbach's α and intraclass correlation coefficient (ICC), respectively. We specifically chose a 2-month interval for test–retest reliability for the present study after considering the acceptable range of time intervals from both methodological (i.e., using a time interval of more than 1 month is not uncommon) (42–44) and logistic (i.e., the participating schools' specific arrangements during the COVID-19 pandemic period) perspectives. Concurrent validity was assessed by bivariate associations of C-IGDC total score and subfactor scores with three gameplay-induced functioning impairment indicators, three aspects of gameplay characteristics (e.g., weekly gameplay frequency), and depressive symptoms.

Following the general evaluation of C-IGDC's psychometric properties, we proceeded to determine an optimal cutoff score to screen for potential IGD cases among Chinese adolescents. With the receiver operating characteristics (ROC) analysis, we first evaluated the general screening efficacy of C-IGDC with the *DSM-5* IGD-referenced area under the curve (AUC). Additional screening efficacy indices were calculated with DAS_STAT (45), which included sensitivity, specificity, positive predictive rate (PPR), negative predictive rate (NPR), Cohen's κ, Youden's index, and diagnostic odds ratio (DOR). In line with Chen et al. (22) and Lowe (46), we took into account all the efficacy indices to identify the optimal point with high sensitivity (increase the detectability of positive cases as high as possible) and relatively high specificity (contain the rate of true negative cases at an acceptable level) among points of sensitivity and specificity levels both higher than 75%. Based on the identified optimal cutoff point, we categorized the overall sample into a probable IGD group and a non-IGD group for between-group comparisons to further evaluate the discriminant power of this proposed point with *t*-test for the continuous variable (i.e., depressive symptoms variable, which was approximately normally distributed in this study), *Mann–Whitney U-*test for those ordinal variables (i.e., gameplay characteristics), and *phi* coefficient (i.e., *r*_φ_) for those nominal variables (i.e., functioning impairment indicators).

## Results

### Factor Structure of C-IGDC in Adolescents

The Chinese adolescent data fitted the proposed two-level, nine-subfactor model well, $\chi_{\left(315\right)}^{2}$ = 1,055.37, *p* < 0.001, CFI = 0.96, TLI = 0.95, RMSEA = 0.043 (90% CI [0.040, 0.046]), SRMR = 0.048. As shown in Table 1 (also see Supplementary Figure S1), each C-IGDC item sufficiently loaded on its corresponding subfactor, with a range of 0.60 to 0.91, which exceeds the minimally expected factor loading of 0.40 (47). Similarly, the nine first-order subfactors adequately loaded on the second-order C-IGDC factor (βs = 0.59–0.94). In contrast, this proposed model also manifested a superior model fit than its alternative single-factor model, which had an unsatisfactory model fit, $\chi_{\left(324\right)}^{2}$ = 3,328.47, *p* < 0.001, CFI = 0.83, TLI = 0.82, RMSEA = 0.086 (90% CI [0.083, 0.089]), SRMR = 0.075.

**Table 1:** Confirmatory factor analysis results of C-IGDC among Chinese adolescents (*N* = 1,253).

**Subconstruct/item**	**Standardized factor loadings**
**F1: Preoccupation**	**0.78** (0.61)
F1A. Preoccupation with Internet games	0.86
F1B. Always anticipate playing Internet games again while not playing	0.79
F1C. Involuntarily imagine things that happened in the Internet games while not playing	0.80
**F2: Withdrawal**	**0.83** (0.69)
F2A: Feel anxious and/or irritated while not being able to play Internet games	0.74
F2B: Feel upset and/or distracted while not being able to play Internet games for any reason	0.82
F2C: Feel like losing everything while not being able to play Internet games	0.91
**F3: Tolerance**	**0.94** (0.87)
F3A: Feel like Internet game is becoming more and more important to you	0.79
F3B: Need to spend more and more time on Internet gaming to feel content	0.81
F3C: Need continued collecting prizes, breaking records, and/or passing more levels to gain desired thrill and/or content	0.60
**F4: Unsuccessful to control**	**0.82** (0.67)
F4A: Have attempted to cut down Internet gaming, but found it difficult	0.82
F4B: Feel like you cannot stop Internet gaming	0.77
F4C: Have attempted to cut down or stop Internet gaming, but failed and started playing again	0.69
**F5: Loss of interests**	**0.66** (0.44)
F5A: Cannot enjoy other activities as much as before because of Internet gaming	0.81
F5B: Decrease the amount of other recreational activities because of Internet gaming	0.74
F5C: Decrease the offline contacts with others because of Internet gaming	0.70
**F6: Continued gaming despite psychosocial problems**	**0.88** (0.78)
F6A: Continued gaming after you understand that Internet gaming has already negatively affected you	0.77
F6B: Continued gaming despite receiving objections from your family	0.75
F6C: Continued gaming for pursuing levels, breaking records, and etc. when you should take a break	0.60
**F7: Deception**	**0.71** (0.50)
F7A: Have deceived others regarding your enthusiasm for Internet gaming	0.65
F7B: Have deceived others regarding your excessive amount of Internet gaming	0.86
F7C: Have deliberately hidden things related to your Internet gaming from others	0.73
**F8: Escape/Relief**	**0.59** (0.35)
F8A: Use of Internet gaming as an escape from the reality	0.75
F8B: Use of Internet gaming to relieve a bad mood	0.83
F8C: Use of Internet gaming to forget your worries	0.84
**F9: Problems**	**0.83** (0.69)
F9A: Conflicts with your family members or friends because of Internet gaming	0.74
F9B: Impaired performance or efficiency at work/study, or even problems, because of Internet gaming	0.64
F9C: Impaired significant aspects in your life because of Internet gaming	0.66

*C-IGDC, Chinese Internet Gaming Disorder Checklist. The second-order standardized factor loadings are bolded. The R^2^ of each latent variable was reported in parentheses*.

### Internal Consistency, Test–Retest Reliability, and Validity

Table 2 showed a high internal consistency for the overall C-IGDC (α = 0.90) and an acceptable range of internal consistency for its nine subfactors (αs =0.60–0.75) in the present adolescent sample. The ICC was 0.83 for the overall C-IGDC scale and ranged from 0.63 to 0.78 for the nine subscales, indicating a good test–retest reliability according to Cicchetti's guideline (48). As for validity, C-IGDC total score showed a moderate, positive correlation with three gameplay-induced functioning indicators (*r*s = 0.30–0.35, *p*s < 0.001), three gameplay characteristics that are related to gameplay amount, frequency, and expense (*r*s = 0.32–0.46, *p*s < 0.001), and depressive symptoms (*r* = 0.32, *p* < 0.001). The nine C-IGDC subfactor scores showed a similar, positive associative pattern with three gameplay-induced functioning indicators (*r*s = 0.15–0.47, *p*s < 0.001), three gameplay characteristics (*r*s = 0.15–0.40, *p*s < 0.001), and depressive symptoms (*r*s = 0.18–0.32, *p*s < 0.001).

**Table 2:** Reliability and bivariate correlations of C-IGDC construct and subconstruct scores (*N* = 1,253).

**Construct/Subconstruct**	***M* (*SD*)**	**C-IGDC**	**F1**	**F2**	**F3**	**F4**	**F5**	**F6**	**F7**	**F8**	**F9**
**C-IGDC** ^**a**^	13.97 (8.78)	(0.90)									
F1: Preoccupation	0.71 (0.50)	0.71***	(0.75)								
F2: Withdrawal	0.26 (0.40)	0.68***	0.45***	(0.72)							
F3: Tolerance	0.54 (0.49)	0.77***	0.56***	0.52***	(0.66)						
F4: Unsuccessful to control	0.53 (0.53)	0.72***	0.44***	0.48***	0.52***	(0.70)					
F5: Loss of interests	0.34 (0.44)	0.60***	0.35***	0.34***	0.35***	0.35***	(0.67)				
F6: Continued playing despite psychosocial problems	0.76 (0.53)	0.76***	0.48***	0.43***	0.54***	0.49***	0.36***	(0.65)			
F7: Deception	0.29 (0.41)	0.61***	0.36***	0.35***	0.36***	0.36***	0.33***	0.37***	(0.66)		
F8: Escape/Relief	0.69 (0.52)	0.62***	0.34***	0.31***	0.45***	0.32***	0.29***	0.39***	0.31***	(0.70)	
F9: Problems	0.52 (0.44)	0.69***	0.38***	0.41***	0.40***	0.43***	0.40***	0.52***	0.41***	0.35***	(0.60)
**Gameplay-induced functioning impairment**											
Physical or mental problems	0.09 (0.29)	0.33***	0.21***	0.23***	0.20***	0.22***	0.22***	0.21***	0.22***	0.22***	0.30***
Relations, study, and/or work impairments	0.22 (0.41)	0.35***	0.15***	0.18***	0.19***	0.27***	0.29***	0.24***	0.23***	0.17***	0.47***
Mental distress	0.08 (0.27)	0.30***	0.19***	0.22***	0.20***	0.18***	0.22***	0.15***	0.18***	0.23***	0.30***
**Gameplay characteristics**											
Weekly gameplay amount	8.57 (9.41)	0.32***	0.29***	0.19***	0.26***	0.24***	0.16***	0.25***	0.15***	0.21***	0.17***
Weekly gameplay frequency	4.02 (1.10)	0.46***	0.40***	0.27***	0.39***	0.38***	0.21***	0.40***	0.23***	0.24***	0.28***
Monthly gameplay expense	1.06 (1.81)	0.35***	0.31***	0.29***	0.31***	0.23***	0.15***	0.25***	0.20***	0.20***	0.19***
**Depressive symptoms**	12.15 (10.95)	0.32***	0.19***	0.24***	0.20***	0.19***	0.19***	0.18***	0.20***	0.32***	0.29***

C-IGDC, Chinese Internet Gaming Disorder Checklist. Cronbach's αs were reported in diagonal parentheses. ***p < 0.001. ^a^The C-IGDC composite score is the summative total of the 27 C-IGDC items, whereas the nine subconstruct scores of F1–F9 are the mean score of the items each subconstruct contains.

### Screening Efficacy and Setting the Optimal Screening Cutoff Point of C-IGDC

With the *DSM-5* IGD cutoff point of ≥5 as a reference, the AUC of the ROC analysis was 0.94 for C-IGDC, indicating a good efficacy. Following the guidelines of Lowe (46), we listed all the five possible cutoff points (i.e., 17–21), with both sensitivity and specificity > 75%, in Table 3. Subsequently, we narrowed down the selection to the points of 20 and 21 because these two had the highest Cohen's κ of 0.59 among the five. Finally, we turned to Youden's index for a higher level of pooled sensitivity and specificity and hence set ≥20 as the optimal screening cutoff point of C-IGDC among adolescent gamers.

**Table 3:** Cutoff points of C-IGDC based on the *DSM-5* proposed diagnostic criteria of IGD (*N* = 1,253).

**Cutoff point (≥)**	**Sensitivity**	**Specificity**	**PPR**	**NPR**	**Cohen's κ**	**Youden's index**	**DOR**
	**(%)**	**(%)**	**(%)**	**(%)**			
16	97.0	73.4	40.9	99.2	0.45	0.70	89.26
17	94.0	76.8	43.5	98.5	0.48	0.71	51.94
18	90.0	81.4	47.9	97.7	0.53	0.71	39.35
19	86.0	85.7	53.3	97.0	0.57	0.72	36.69
**20**	**81.0**	**88.1**	**56.4**	**96.1**	**0.59**	**0.69**	**31.65**
21	75.5	90.0	59.0	95.1	0.59	0.66	27.82
22	72.5	92.4	64.4	94.6	0.62	0.65	32.07

*C-IGDC, Chinese Internet Gaming Disorder Checklist; DSM-5, the fifth edition of the Diagnostic and Statistical Manual of Mental Disorders; IGD, Internet Gaming Disorder; PPR, Positive Predictive Rate; NPR, Negative Predictive Rate; DOR, Diagnostic Odds Ratio. The line in bold indicates the optimal screening cutoff score*.

To further examine the discriminant power of this optimal cutoff point, we applied this cutoff point and divided our overall sample into probable IGD gamers (22.9%) and non-IGD gamers (77.1%) for further comparison. Compared to non-IGD gamers, probable IGD gamers reported significantly more depressive symptoms [*t*_(427)_ = 8.49, *p* < 0.001] and more functioning impairments in aspects of physical or mental health (*r*_φ_ = 0.23, *p* < 0.001), relations, study, and/or work (*r*_φ_ = 0.25, *p* < 0.001), and mental distress (*r*_φ_ = 0.22, *p* < 0.001), as well as being more involved in gaming in terms of more weekly gameplay amount (*U* = 83,370.5, *p* < 0.001), higher weekly gameplay frequency (*U* = 78,714.0, *p* < 0.001), and more monthly gameplay expense (*U* = 94,913.5, *p* < 0.001).

## Discussion

This study showed that IGD symptoms were not uncommon among Chinese adolescents and provided empirical support to apply the 27-item C-IGDC to this population for measuring IGD severity and screening for probable IGD cases. Consistent with previous findings in Chinese adults (22), our adolescent past-year gamer data supported the conceptualized two-level, nine-subfactor model structure for C-IGDC. High factor loadings were found across all nine first-order subconstructs on the second-order C-IGDC construct (βs = 0.59–0.94), lending extra support to the good fit between the data and the proposed model structure. Similar to how C-IGDC performed in the Chinese adult sample (22), the first-order *Deception* (F7) and *Escape/Relief* (F8) presented a relatively lower factor loading, compared to other subfactors, on the parent second-order C-IGDC construct; this pattern has also been found in other *DSM-5* based assessment tools for IGD [e.g., (49, 50)]. The relatively more minor contribution of *Loss of interests* (F5) to C-IGDC was only found in the present Chinese adolescents but not in the previous Chinese adult sample. This discovery may indicate that loss of interest is less typically observed in probable IGD cases among Chinese adolescents than their adult counterparts. One plausible reason is the relatively higher proportion of online gaming observed in Chinese adolescents (e.g., 64.7% in middle school netizens) than the general Chinese population (e.g., 58.9% in the overall netizens) in recent years (51, 52), implying a lower baseline level of other interests since the beginning.

For reliability, the overall C-IGDC scale displayed high internal consistency (α = 0.90) and good 2-month test–retest reliability (ICC = 0.83) in the present Chinese adolescent sample, and the former was comparable to that reported in the previous Chinese adult sample (α = 0.92) (22). Five out of the nine C-IGDC subfactors (i.e., *Tolerance* [F3], *Loss of interests* [F5], *Continued playing despite psychosocial problems* [F6], *Deception* [F7], and *Problems* [F9]) presented an internal consistency coefficient between 0.60 and 0.70, which is lower than the rule of thumb of good reliability as 0.70 or higher but still within the acceptable range provided that the two-level, nine-subfactor model of C-IGDC showed a good construct validity (i.e., βs >0.50) (53). The concurrent validity of C-IGDC in adolescents was supported by moderately positive associations (*r*s = 0.30–0.46, *p* < 0.001) of C-IGDC with functioning impairments (i.e., physical or mental problems, impairments in relations, study, and/or work, and mental distress), gameplay involvements (i.e., amount, frequency, and expense), and depressive symptoms. These associative patterns were also consistent with previous studies that averagely small-to-medium effect size was found for the positive correlation between disordered gaming and adverse health outcomes (23, 24), whereas there was an at least moderate-level link of IGD to depression (26, 31). Extant research also revealed that probable IGD gamers tended to spend significantly more time and money on gaming (9, 26, 27), corresponding to the moderately positive association between C-IGDC and gameplay involvement observed in the current study.

C-IGDC also demonstrated a satisfactory overall screening efficacy (AUC = 0.94) in identifying probable IGD cases that fit the *DSM-5* proposed diagnostic criteria in Chinese adolescents. Based on our adolescent sample, the exact optimal screening cutoff point of ≥20 was suggested for the Chinese young adult sample (22). Using this cutoff point of ≥20, C-IGDC had 81.0% sensitivity and 88.1% specificity in Chinese adolescents; these statistics were comparable to those reported in a Chinese adult sample (81.7% sensitivity and 85.4% specificity) (22) and those shown by other screening tools for Internet-related disorders developed in Chinese samples, such as Ko et al.'s (54) Chen Internet Addiction Scale (83.9% sensitivity and 76.7% specificity) and its subsequent Chen Internet Addiction Scale—Gaming Version (97.1% sensitivity and 76.8% specificity) (55). In the present past-year adolescent gamer sample, the probable IGD cases, identified by C-IGDC and the *DSM-5* diagnostic criteria, were 22.9% and 16.0%, respectively; compared to the corresponding prevalence estimates of 23.3% and 12.9% in the former young adult sample (22), we spotted a noticeable raise in the specificity level of C-IGDC's proposed screening cutoff score for Chinese adolescents. Moreover, C-IGDC-identified probable IGD gamers reported significantly higher levels of functioning impairments, depressive symptoms, and gameplay involvement in terms of time and money, lending additional information on a good discriminant power of C-IGDC for Chinese adolescent gamers.

The current study design was limited in several aspects. First, we could not evaluate the predictive validity of C-IGDC with the current sample because of its cross-sectional nature. We hope this missing piece of information can be addressed by subsequent analyses using longitudinal data. Second, although we have extended the application of C-IGDC from adults to adolescents in the present study, there is still a gap for its use among children, given a high and increasing proportion of low-age gamers in recent years. For example, a recent report on Internet use among minors in China found that gamers took up 56.7% of primary school netizens and 64.7% of middle school netizens (51). A follow-up validation of C-IGDC among the younger population can promote an earlier screening for high-risk IGD cases and allow efficacy evaluation of those more timely psychoeducational campaigns to foster their balanced work-and-play lifestyle. Third, data collected from the survey method is probably subjected to self-report bias to a certain extent. Ideally, future studies can acquire supplementary behavioral data or third-party data from parents or friends for comparison.

To conclude, as the first *DSM-5* based IGD screening tool stemmed from the Chinese population, C-IGDC exhibited adequate psychometric soundness for Chinese adolescent gamers with past-year gaming experience in terms of satisfactory results of model fit between the data and the proposed two-level model structure, internal consistency, test–retest reliability, concurrent validity, and screening efficacy. The proposed screening cutoff score of ≥20 could effectively detect probable IGD cases among Chinese adolescent past-year gamers in the present study, indicating its potential to facilitate health professionals' work during mass screening for high-risk IGD gamers at the school and clinical settings. In practice, users are advised to administrate a pre-screening item regarding respondents' past-year gaming frequency and only invite those who have gaming engagement in the past 12 months for a C-IGDC screening to increase the cost-effectiveness for IGD screening. Future studies are also recommended to extend the use of C-IGDC to a two-stage epidemiological evaluation of Chinese IGD, which involves applying C-IGDC to screen for probable IGD adolescent cases in the first stage and performing clinical diagnostic interviews in the second stage to identify a diagnostic cutoff point of C-IGDC to facilitate the IGD diagnostic efficiency.

## Data Availability Statement

The raw data supporting the conclusions of this article will be made available by the authors, without undue reservation.

## Ethics Statement

The studies involving human participants were reviewed and approved by Research Committee-Panel on Research Ethics, University of Macau. Written informed consent from the participants' legal guardian/next of kin was not required to participate in this study in accordance with the national legislation and the institutional requirements.

## Author Contributions

JC: project administration, methodology, investigation, formal analysis, writing—original draft, and writing—reviewing and editing. XS: investigation, resources, and writing—original draft. LD: resources and writing—reviewing and editing. AW: conceptualization, funding acquisition, supervision, and writing—reviewing and editing. All authors contributed to and approved the final manuscript.

## Funding

This study was supported by research grants of the corresponding authors' affiliated institution, the University of Macau (MYRG2019-00014-FSS and CRG2020-00001-ICI).

## Conflict of Interest

The authors declare that the research was conducted in the absence of any commercial or financial relationships that could be construed as a potential conflict of interest.

## Publisher's Note

All claims expressed in this article are solely those of the authors and do not necessarily represent those of their affiliated organizations, or those of the publisher, the editors and the reviewers. Any product that may be evaluated in this article, or claim that may be made by its manufacturer, is not guaranteed or endorsed by the publisher.

## Footnotes

^1^Included regional data from Singapore (8.7–17.7%) and Thailand (5.4–7.5%).

^2^Included regional data from Australia (5.1%), China (3.4–3.9%), Finland (9.1%), Germany (1.2–5.2%), Greece (1.4%), Hong Kong (15.6%), Hungary (4.6%), Iran (5.3%), Lithuania (9.2%), Japan (5.5%), Korea (3.5–5.9%), Netherlands (1.6–4.0%), Norway (0.9%), Singapore (7.6%), Slovenia (2.5%), Switzerland (2.3%), Taiwan (8.1%), the USA (4.9-8.5%), and cross-country studies in Europe and/or Northern America (0.7–3.6%).

^3^Included regional data from Australia (1.8–7.0%), Canada (9.4%), England (6.0–19.9%), Germany (1.2–8.9%), Great Britain (14.6%), Greece (2.5%), Hong Kong (15.7%), Hungary (4.6%), Iceland (1.8%), Iran (5.3%), Netherlands (1.0%), Poland (2.0%), Spain (0.6–7.7%), Sweden (1.3–2.4%), and Romania (1.3%).
