# Supplementary material for: Evaluation of the Psychometric Properties of the Chinese Internet Gaming Disorder Checklist (C-IGDC) Among Chinese Adolescents
Source: Front Psychiatry. 2021 Sep 13;12:721397. doi: 10.3389/fpsyt.2021.721397 (PMC8473869; doi:10.3389/fpsyt.2021.721397)
Supplement: Supplementary file 1 [file Data_Sheet_1.pdf]

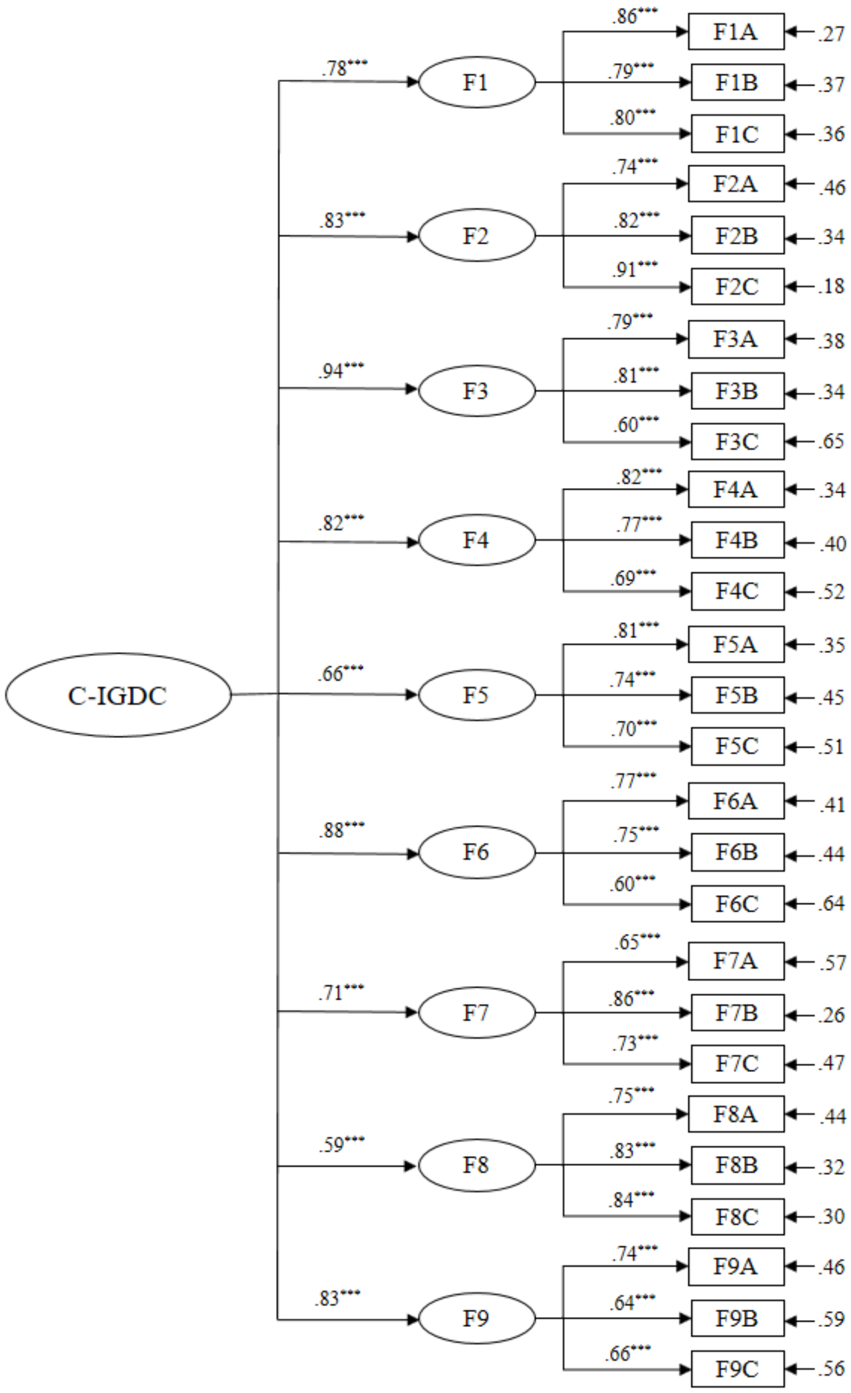

Figure S1. Two-level, nine-subfactor structure of the 27-item Chinese Internet Gaming Disorder Checklist (C-IGDC) among Chinese past-year adolescent gamers ($N = 1,253$). Factor loadings are standardized scores. F1 = *Preoccupation*, F2 = *Withdrawal*, F3 = *Tolerance*, F4 = *Unsuccessful to control*, F5 = *Loss of interests*, F6 = *Continued gaming despite psychosocial problems*, F7 = *Deception*, F8 = *Escape/Relief*; F9 = *Problems*. See Table 1 and Table S1 for detailed descriptions of the C-IGDC items in English and Chinese, respectively.

Table S1. The Chinese Internet Gaming Disorder Checklist (C-IGDC) items in Chinese characters (華人網絡遊戲成癮篩查量表)

題號	請問在過去的 12 個月，以下情況有多經常發生在你身上? 請在下表中圈出對應的頻率，其中 0 = 從不，1 = 有時，2 = 常常。	從不	有時	常常
F1A	你腦海裡會充滿著關於網絡遊戲的事情嗎?	0	1	2
F1B	你在不玩網絡遊戲的時候，會總惦記著下次再玩嗎?	0	1	2
F1C	當你不玩網絡遊戲的時候，你會不由自主地想象網絡遊戲中發生的事嗎?	0	1	2
F2A	你在不能玩網絡遊戲的時候，會感覺比較急躁易怒嗎?	0	1	2
F2B	不論因為任何原因不能玩網絡遊戲的時候，你會覺得比較心煩意亂嗎?	0	1	2
F2C	當你無法玩網絡遊戲的時候，你會感到像失去了所有或全部嗎?	0	1	2
F3A	你會感到網絡遊戲對你越來越重要嗎?	0	1	2
F3B	你會需要用越來越多的時間玩網絡遊戲才能滿足嗎?	0	1	2
F3C	你會需要不斷收集、打破記錄，或過關來獲得嚮往的興奮或滿足感嗎?	0	1	2
F4A	你試過想少玩點網絡遊戲，但感到很難做到嗎?	0	1	2
F4B	你會覺得你沒法不玩網絡遊戲嗎?	0	1	2
F4C	你試過減少甚至停止玩網絡遊戲，但之後又開始玩嗎?	0	1	2
F5A	網絡遊戲會使你不再像以前一樣享受其他活動嗎?	0	1	2
F5B	你會因為網絡遊戲而減少參與其他消遣活動嗎?	0	1	2
F5C	你會因為玩網絡遊戲而減少了和別人的線下互動嗎?	0	1	2
F6A	你會在明白玩網絡遊戲對你有負面影響之後，繼續玩網絡遊戲嗎?	0	1	2
F6B	即使家人反對，但你還是會繼續玩網絡遊戲嗎?	0	1	2
F6C	你試過到了應該休息的時間， 但為了想過關或破紀錄等原因而繼續玩網絡遊戲嗎?	0	1	2
F7A	你試過對其他人欺瞞你對網絡遊戲的熱衷程度嗎?	0	1	2
F7B	你試過因為玩網絡遊戲太多而對其他人說謊嗎?	0	1	2
F7C	你會刻意對其他人隱瞞你玩網絡遊戲的一些事嗎?	0	1	2
F8A	你會用玩網絡遊戲來逃避現實生活嗎?	0	1	2
F8B	你會用玩網絡遊戲來處理你的壞情緒嗎?	0	1	2
F9C	你會藉玩網絡遊戲來忘掉煩憂嗎?	0	1	2
F9A	你試過因為玩網絡遊戲而與家人或朋友發生爭執或衝突嗎?	0	1	2
F9B	玩網絡遊戲會令你在工作(或學業)上的表現或效率變差，甚至出現問題嗎?	0	1	2
F9C	玩網絡遊戲會對你生活裡重要的事情產生負面影響嗎?	0	1	2

Note. F1-*Preoccupation* (因子 1-沈湎遊戲): F1A, F1B, F1C, F2-*Withdrawal* (因子 2. 遊戲戒斷): F2A, F2B, F2C; F3-*Tolerance* (因子 3-遊戲耐受): F3A, F3B, F3C; F4-*Unsuccessful to control* (因子 4-控制失敗): F4A, F4B, F4C; F5-*Loss of interests* (因子 5-興趣減少): F5A, F5B, F5C; F6- *Continued playing despite psychosocial problems* (因子 6-明知故玩): F6A, F6B, F6C; F7-*Deception* (因子 7-隱瞞欺騙): F7A, F7B, F7C; F8-*Escape/Relief* (因子 8-情緒解脫): F8A, F8B, F8C; F9-*Problems* (因子 9-問題行為): F9A, F9B, F9C.
